# Modulation of Retrograde Trafficking of KCa3.1 in a Polarized Epithelium

**DOI:** 10.3389/fphys.2017.00489

**Published:** 2017-07-18

**Authors:** Bob Shih-Liang Lee, Daniel C. Devor, Kirk L. Hamilton

**Affiliations:** ^1^Department of Physiology, School of Biomedical Sciences, University of Otago Dunedin, New Zealand; ^2^Department of Cell Biology, University of Pittsburgh School of Medicine Pittsburgh, PA, United States

**Keywords:** K^+^ channels, ubiquitylation, deubiquitylation, DCEBIO, clotrimazole

## Abstract

In epithelia, the intermediate conductance, Ca^2+^-activated K^+^ channel (KCa3.1) is targeted to the basolateral membrane (BLM) where this channel plays numerous roles in absorption and secretion. A growing body of research suggests that the membrane resident population of KCa3.1 may be critical in clinical manifestation of diseases. In this study, we investigated the key molecular components that regulate the degradation of KCa3.1 using a Fisher rat thyroid cell line stably expressing KCa3.1. Using immunoblot, Ussing chamber, and pharmacological approaches, we demonstrated that KCa3.1 is targeted exclusively to the BLM, provided a complete time course of degradation of KCa3.1 and degradation time courses of the channel in the presence of pharmacological inhibitors of ubiquitylation and deubiquitylation to advance our understanding of the retrograde trafficking of KCa3.1. We provide a complete degradation profile of KCa3.1 and that the degradation is via an ubiquitin-dependent pathway. Inhibition of E1 ubiquitin activating enzyme by UBEI-41 crippled the ability of the cells to internalize the channel, shown by the increased BLM surface expression resulting in an increased function of the channel as measured by a DCEBIO sensitive K^+^ current. Additionally, the involvement of deubiquitylases and degradation by the lysosome were also confirmed by treating the cells with PR-619 or leupeptin/pepstatin, respectively; which significantly decreased the degradation rate of membrane KCa3.1. Additionally, we provided the first evidence that KCa3.1 channels were not deubiquitylated at the BLM. These data further define the retrograde trafficking of KCa3.1, and may provide an avenue for therapeutic approach for treatment of disease.

## Introduction

The intermediate-conductance, Ca^2+^-activated K^+^ channel, KCa3.1 (KCNN4), was first described as a channel of predominately epithelial tissues (Ishii et al., 1997; Joiner et al., 1997; Logsdon et al., 1997). Now, however, KCa3.1 has been demonstrated in a range of tissues including endothelia, nervous, and smooth and cardiac muscle tissues with numerous cellular functions (Neylon et al., 1994, 1999, 2004; Köhler et al., 2000; Chachi et al., 2013; Weisbrod et al., 2013; Turner et al., 2015). With respect to epithelia, KCa3.1 was first described in the basolateral membrane (BLM) of epithelial cells with electrophysiological and pharmacological approaches (McCann and Welsh, 1990; Devor and Frizzell, 1993; Hamilton et al., 1999; Hamilton and Keissling, 2006). KCa3.1 has numerous cellular functions, of which, one is that the activity of basolateral KCa3.1 leads to a hyperpolarization of the cellular membrane potential, thus, providing a driving force for ion and fluid transport across the epithelial cell (e.g., Cl^−^ secretion and Na^+^ absorption; Balut et al., 2012; Devor et al., 2016).

A growing body of evidence suggests that the membrane surface KCa3.1 may be crucial in clinical manifestation of diseases. For example, in ulcerative colitis (UC), the number of KCa3.1 channels present at the plasma membrane is reduced while membrane KCa3.1 channels are increased in polycystic kidney disease (PKD) resulting in increased fluid in patients with PKD (Albaqumi et al., 2008; Al-Hazza et al., 2012). Recently, Rapetti-Mauss et al. (2015) and others (Andolfo et al., 2015; Glogowska et al., 2015) identified specific “gain of function” mutations of KCa3.1 that are associated with chronic hemolysis (hereditary xenocytosis) characterized by red blood cell dehydration and osmotic fragility. These were the first reports of a human disease attributed directly to a mutation in KCa3.1.

Clearly, understanding how one can modify/modulate the membrane population of KCa3.1 can alter physiological function. Therefore, in light of these clinical examples, a thorough understanding of the trafficking of KCa3.1 to (e.g., anterograde) and from (e.g., retrograde) the plasma membrane remains crucial.

In the past, specific leucines of the leucine zipper motifs of the C- and N termini of KCa3.1 have been mutated to alanine resulting in altered assembly and trafficking of the channel to the plasma membrane of human epithelial kidney (HEK293) cells (Syme et al., 2003; Jones et al., 2004, 2005). Additionally, Balut et al. (2010a), using a novel-tagged channel approach reported, in non-polarized HEK293 cells, that KCa3.1 was endocytosed from the plasma membrane in <90 min and targeted, via a Rab7-ESCRT (endosomal sorting complexes required for transport)-dependent mechanism, for degradation by the lysosome. Subsequently, Balut et al. (2011) demonstrated that KCa3.1 was mildly ubiquitylated at the plasma membrane and polyubiquitylated following endocytosis and targeted to the lysosome where upon UPS8 (ubiquitinin specific protease) altered the final rate of delivery to the lysosome by deubiquitylating KCa3.1 prior to its degradation.

These past studies have examined the trafficking of KCa3.1 in non-polarized epithelia. However, at present, there still is a significant gap in our understanding of the trafficking of KCa3.1 in polarized epithelial cells. Bertuccio et al. (2014) reported that anterograde trafficking and maintenance of KCa3.1 at the BLM of polarized epithelial cells were dependent upon Rab1 and Rab8. Recently, we demonstrated that the targeting of KCa3.1 to the BLM of epithelial cells was a cytoskeletal- and myosin-Vc-dependent process (Farquhar et al., 2017). Further, Bertuccio et al. (2014) have confirmed in polarized epithelia that the KCa3.1 was ubiquitylated prior to and after endocytosis, inhibition of ubiquitylation reduced degradation, and that the trafficking of KCa3.1 was independent of recycling endosomes, and did not require the AP-1 adaptor protein, μ1B for proper trafficking (Bertuccio et al., 2014).

The aim of our study was to further examine, using a pharmacological approach, the time course of degradation of KCa3.1, to ascertain whether modulation of ubiquitylation and deubiquitylation alters the physiological function of KCa3.1 at the BLM and/or the post-endocytic degradation of KCa3.1 of polarized epithelial cells.

## Materials and methods

### Molecular biology

The biotin ligase acceptor peptide (BLAP) sequence (GLNDIFEAQKIEWHE) was inserted into the extracellular loop of KCa3.1 between the transmembrane domains S3 and S4 as previously described (Balut et al., 2010a; Gao et al., 2010; Farquhar et al., 2017). KCa3.1-BLAP and BirA (biotin ligase) with an endoplasmic reticulum (ER) retention sequence, KDEL (kindly provided by Dr. Alice Ting, Massachusetts Institute of Technology, Cambridge, MA, Chen et al., 2005; Howarth and Ting, 2008), were subcloned into a bicistronic plasmid, pBudCE4.1 (Invitrogen, Carlsbad, CA, USA) behind the EF-1α and CMV promoters, respectively (Balut et al., 2010a,b, 2011; Gao et al., 2010).

### Cell culture and fischer thyroid rat cell line stably KCa3.1-BLAP and BirA-KDEL

Fischer rat thyroid (FRT) cells were used as a cell model of a polarized epithelium (Zurzolo et al., 1991). A stable FRT cell line was generated by transfecting in the bicistronic plasmid expressing both KCa3.1-BLAP and BirA-KDEL into FRT cells using Lipofectamine 2000 (Invitrogen) following the manufacturer's instructions and selecting a stable cell line using zeocin (850 μg/ml; Winter et al., 2011). We have previously demonstrated that insertion of the BLAP sequence into KCa3.1 did not affect the Ca^2+^ sensitivity, activation by DCEBIO, or the inhibition by clotrimazole (Gao et al., 2010). Both untransfected FRT and the KCa3.1-BLAP + BirA-KDEL FRT cell lines were cultured in Nutrient Mixture F-12 (Ham's F-12; Invitrogen) at a pH of 7.4, followed by the addition of 10% fetal bovine serum and 1% penicillin–streptomycin. Cells were grown in 25 mm^2^ flasks (NUNC, ThermoFisher Scientific, Waltham, MA, USA) and incubated in a humidified 5% CO_2_, 95% O_2_ incubator at 37°C. For all experiments, untransfected and stably expressing cells were grown (72 h) on Transwell™ or Snapwell™ permeable support filters (Corning, NY) to establish a polarized epithelium and allowing access to both apical and basolateral sides of the epithelium. All experiments were conducted with cells from passage numbers 10–20 for this study. The University of Otago Institutional Biological Safety Committee approved all experimental protocols and the molecular biological techniques used in this study.

### Biotinylation and streptavidin labeling of KCa3.1-BLAP

Within the stable KCa3.1-BLAP BirA-KDEL cell line, the BirA-KDEL is retained within the ER. Therefore, after assembled, KCa3.1-BLAP is biotinylated in the ER prior to being trafficked out to the plasma membrane. Streptavidin labeling of surface KCa3.1-BLAP was performed as we have previously described (Balut et al., 2010a; Gao et al., 2010; Bertuccio et al., 2014; Farquhar et al., 2017). Briefly, upon reaching confluence (72 h after cell seeding), cells were taken out of the incubator for labeling; all procedures and solutions were maintained at 4°C to prevent channel internalization. Cells were first washed with 2 ml of 4°C PBS with 1% bovine serum albumin (BSA) on both apical and basolateral sides of the permeable support filter to eliminate residual media. Since streptavidin is cell impermeable, the cells were labeled by applying streptavidin (10 μg/ml of streptavidin in PBS with 1% BSA) on the desired side (apical or basolateral, or both) for 30 min at 4°C. After labeling, cells were washed three times with PBS with 1% BSA and three times with PBS to eliminate residue streptavidin and incubated for various periods of time at 37°C as indicated in the text (Balut et al., 2010a; Bertuccio et al., 2014; Farquhar et al., 2017). Generally, for a given experiment, stably transfected FRT cells were grown on filters for 72 h, channels were labeled basolaterally with streptavidin followed by immediate lysis of cells for *t* = 0, or filters were returned to the incubator for varying incubation times (1, 3, 5, 8, or 12 h in 37°C) in the presence of a pharmacological inhibitor followed by IB.

### Immunoblot experiments

Immunoblot (IB) experiments were performed as described previously (Jones et al., 2004, 2007; Balut et al., 2010a,b; Gao et al., 2010; Bertuccio et al., 2014; Farquhar et al., 2017). Briefly, cells were lysed and protein concentrations were determined by the BCA protein assay (Walker, 1994). Equal amounts of protein (30 μg) were loaded into wells of a gel (6 or 8%) and protein standard (8 μl) used (BenchMark™ pre-stained protein ladder; Invitrogen, Cat No. 10748-010) and resolved with SDS-PAGE for 150 mV for 90 min (Hoefer Mighty Small II system, Cat. No. 80-6149-35, Amersham Biosciences Corp. Piscataway, NJ, USA). Proteins were transferred (50 V, 2 h) with a semi-dry transfer unit (Hoefer, EPS 2A200) to polyvinylidene difluoride (PVDF) membranes for further IB analysis with α-streptavidin antibody. Proteins bands were visualized by enhanced chemiluminescence detection (Lumilight, Roche, Basel Switzerland). Blots were probed for β-actin as a protein loading control. The bands obtained from immunoblot analysis were quantified by densitometry, using the GS-700 densitometer (Bio-Rad) and the Quantity One programme (BioRad laboratories). The obtained band intensities for the various time points were normalized to β-actin and then compared relative to the intensity at time 0 (*t* = 0) and reported.

### Antibodies

Polyclonal rabbit α-streptavidin IgG antibody (1:2,000, Genscript, Piscataway, NJ, USA) was used to detect streptavidin-labeled membrane bound KCa3.1-BLAP. Mouse monoclonal β-actin IgG antibody (1:10,000, Sigma-Aldrich, New Zealand) was used to detect β-actin for the immunoblot experiments. Secondary antibodies used included goat anti-rabbit conjugated to horseradish peroxidase (HRP; 1:2,000, GE Life Science, New Zealand) and HRP conjugated goat anti-mouse antibody (1:2,000, Sigma-Aldrich).

### Ussing chamber experiments

Ussing chamber experiments were conducted to examine the effect of inhibitors on the functional expression of KCa3.1, as measured as K^+^ currents (I_K_). I_K_ was measured by a VCC MC Ussing chamber system that consisted of an Easymount chamber system and an 8-channel voltage/current clamp unit (Physiologic Instruments, San Diego, CA, USA) as previously described (Farquhar et al., 2017). FRT cells were grown on Snapwell™ filters for 3–5 days prior to an experiment and exposed to an inhibitor for X h (mucosa, m and serosal, s) prior to the experiments (details are stated in the text). Once a filter was mounted into a chamber, the apical (muosal) surface of the monolayer was bathed in a solution containing (in mM) 145 potassium gluconate, 10 HEPES, 1 MgCl, 4 CaCl_2_, and 10 glucose (pH 7.4) and the basolateral (serosal) surface was bathed in a solution containing (in mM) 140 sodium gluconate, 5 potassium gluconate, 10 HEPES, 1 MgCl, 4 CaCl_2_, and 10 glucose (pH of 7.4). All solutions were maintained at 37°C. The CaCl_2_ was increased from the normal 1.2 to 4 mM to compensate for the Ca^2+^-buffering capacity of the gluconate anion (Durham, 1983). To assess the effect of drugs on the targeting of KCa3.1 to the BLM, I_K_ via KCa3.1 was measured which consisted of stimulation of KCa3.1 with the addition of DCEBIO (100 μM, m and s), a KCa3.1 specific activator (Singh et al., 2001), and inhibiting the KCa3.1 stimulated current by the addition of clotrimazole (10 μM, m and s; Devor et al., 1997). Therefore, using the combination of DCEBIO and clotrimazole allowed the determination of the effect of inhibitors on the targeting of KCa3.1 to the BLM as measured by I_K_. Stably-transfected FRT cells not incubated with inhibitors served as positive controls while wild-type non-transfected FRT cells served as negative controls for the Ussing experiments.

### Chemicals

All chemicals were purchased from Sigma-Aldrich, unless otherwise stated. PR-619 was purchased from Life Sensors (Malvern, PA). UBEI-41 was purchased from BioGenova (Potomac, MD). DMSO was used as a vehicle for DCEBIO, clotrimazole, UBEI-41, and PR-619.

### Statistical analyses

The density data of the immunoblots were analyzed by the programs Excel 2007 (Microsoft, Redmond, WA, USA) and Prism 5 (Graph Pad Software, La Jolla, CA, USA). To compare the normalized values of the IB band intensities, statistical analysis was performed using the non-parametric Kruskal–Wallis test followed by a with Dunn's post-test. For the Ussing chamber experiments, the recorded traces were analyzed by the program Excel 2007 (Microsoft). Student's unpaired *t*-test was used to compare control and the UBEI-41 treated samples of the same time point, for example. One-way ANOVA with a Dunnett's post-test was conducted for the comparison of multiple time points. All data are presented as means ± standard error of the mean (S.E.M.) and where *n* indicates the number of experiments from different passages of cells. Values of *P* ≤ 0.05 were considered statistically significant. All experiments were repeated from different passages of cell at least three times to ensure the fidelity of the results.

## Results

### Membrane location of KCa3.1 and time course of degradation of KCa3.1

**Figure 1:**
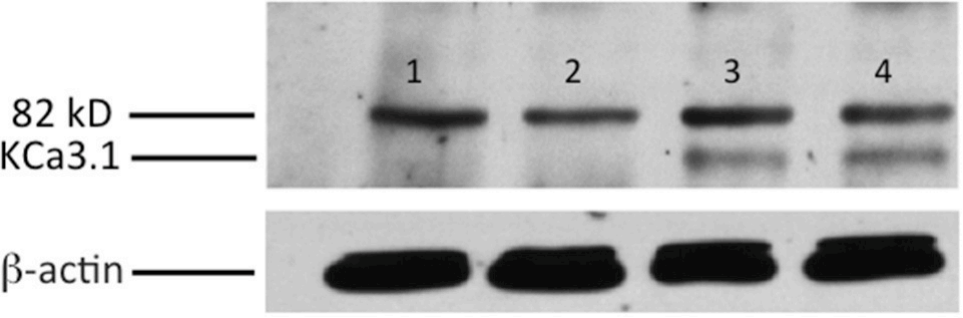
Membrane location of KCa3.1-BLAP stably expressed in FRT cells. The sidedness experiments confirm the expression pattern of KCa3.1 on the basolateral membrane of FRT cells. FRT cells with KCa3.1-BLAP were grown on Transwell™ filter inserts to form a monolayer, and then labeled with streptavidin in the apical, basolateral, or both sides of the filter insert. Bands were only observed in the samples that were labeled basolaterally near the 82 kD mark. Lane 1: untransfected FRT, Lane 2: KCa3.1-BLAP + Apical streptavidin labeling, Lane 3: KCa3.1 + Basolateral streptavidin labeling, Lane 4: KCa3.1 + Apical and Basolateral streptavidin labeling. Each lane was loaded with 30 μg of protein and β-actin was used as a loading control (*n* = 5).

Initially, we confirmed the membrane location of the KCa3.1-BLAP channel of our stably transfected FRT cell line. As seen in Figure 1, KCa3.1 was highly expressed in the BLM of the polarized epithelial cells (*n* = 5). These results confirm previous findings of Bertuccio et al. (2014) and Farquhar et al. (2017).

**Figure 2:**
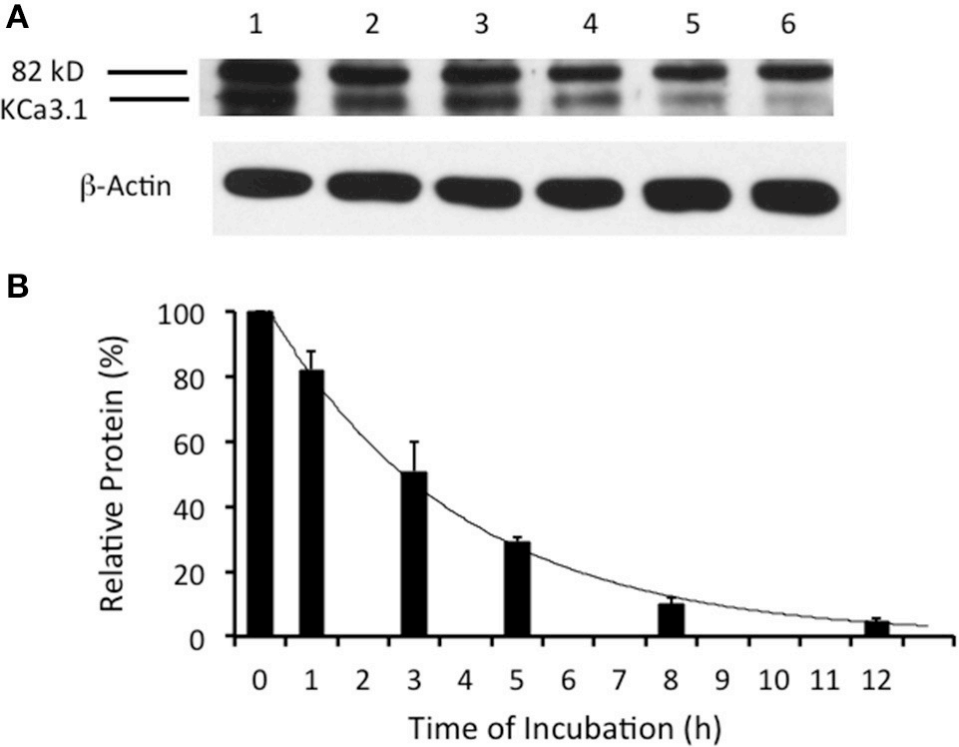
Degradation time course of membrane labeled KCa3.1-BLAP channels. Time course of KCa3.1 was investigated over a 12 h period. **(A)** After basolateral streptavidin labeling, cells were further incubated in 37°C for 0 (no incubation), 1, 3, 5, 8, or 12 h before being lysed and detection of KCa3.1 via immunoblot. Lane 1: Control no incubation (*t* = 0), Lane 2: 1 h of incubation (*t* = 1), Lane 3: 3 h of incubation (*t* = 3), Lane 4: 5 h of incubation (*t* = 5), Lane 5: 8 h of incubation (*t* = 8), and Lane 6: 12 h of incubation (*t* = 12) **(B)** Immunoblot results were quantified by densitometry. Relative protein expression demonstrated that the half-life of KCa3.1 was ~3 h with only 5% of the labeled channels remaining at 12 h. Each lane was loaded with 30 μg of protein and β-actin was used as a loading control (*n* = 8).

The time course of degradation of KCa3.1 differs widely depending upon epithelial cell types as the half-life of KCa3.1 in heterologous expression systems (HEK293 cells, MDCK cells, and Caco-2 cells) varies between 3 and 16 h with cells grown on plastic or permeable supports (Jones et al., 2004, 2005; Gao et al., 2008, 2010; Balut et al., 2010b). Recently, it was reported that the time course of degradation of KCa3.1 was ~3 h for FRT polarized cells (Bertuccio et al., 2014). Here, we confirm those findings to establish a control time course data set that was used to compare experimental results for inhibitors of retrograde of KCa3.1 (sections presented below). It should be noted that even though the channels were labeled at the BLM, the protein expression level derived from the IB at *t* = 0 h is only labeled basolateral surface membrane channels. As exhibited in Figure 2, KCa3.1-BLAP was significantly (*P* ≤ 0.05) degraded over 12 h of incubation with a channel half-life of ~3 h and only 4 ± 2% of the originally labeled channel remained within the cell after 12 h (*P* ≤ 0.05, *n* = 8).

### Ubiquitylation of KCa3.1 in a polarized epithelium

**Figure 3:**
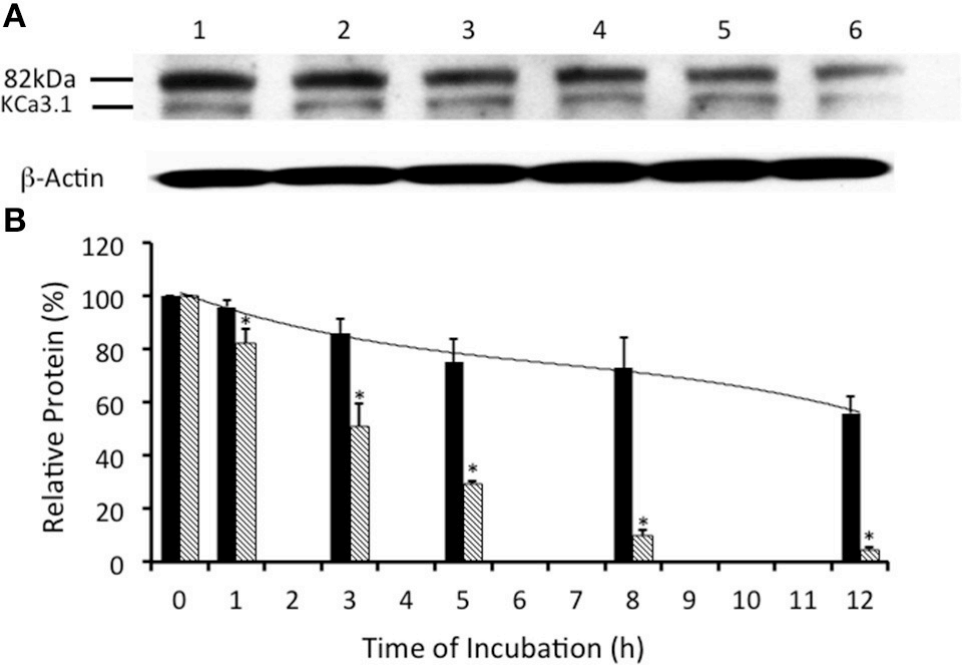
Effects of E1-ubiquitin activating enzyme inhibition via UBEI-41 on the expression of streptavidin labeled KCa3.1-BLAP channels. **(A)** After basolateral streptavidin labeling of KCa3.1 channels, cells were further incubated in 37°C for 0 (no incubation), 1, 3, 5, 8, or 12 h with UBEI-41 (50 μM) before being lysed and detection of KCa3.1 by immunoblot. Lane 1: Control no incubation (*t* = 0), Lane 2: 1 h of incubation (*t* = 1), Lane 3: 3 h of incubation (*t* = 3), Lane 4: 5 h of incubation (*t* = 5), Lane 5: 8 h of incubation (*t* = 8), and Lane 6: 12 h of incubation (*t* = 12). Each lane was loaded with 30 μg of protein and β-actin was used as a loading control. **(B)** Relative expression levels of KCa3.1 in the presence of UBEI-41 (black bars). Relative expression levels of KCa3.1 (serving as control data) from Figure 2 were plotted (hatched bars). Relative protein expression demonstrated that the expression level of KCa3.1 (control, 0 h, black bar) remained the same after 1 h of treatment of UBE-41, but was higher than the 1 h control non-treated cells (hatched bars). Also, by 3 h only ~10% of the labeled channel had been degraded compared with the 0 h control, however, at 3 h, the non-treated cells exhibited degradation of ~50% of the labeled KCa3.1 (hatched bar) (^*^P ≤ 0.05, *n* = 4). Thus, UBEI-41 effectively slowed the degradation of KCa3.l, extending the channel half-life to more than 12 h compared to non-treated control cells (~3 h).

Many surface membrane proteins require ubiquitylation prior to endocytosis (Rotin and Staub, 2011; Neutzner and Neutzner, 2012). Indeed, Balut et al. (2011) and Bertuccio et al. (2014) reported that KCa3.1, expressed in non-polarized HEK293 cells or polarized Caco-2 cells, was ubiquitylated at the plasma membrane and further polyubiquitylation occurred after endocytosis. To determine whether KCa3.1 is ubiquitylated in a similar manner in FRT cells, we used UBEI-41 that is a cell permeable inhibitor of the E-1 ubiquitin-activating enzyme (Yang et al., 2007). This enzyme is the first enzyme in a cascade that results in the ubiquitylation of proteins, which are targeted to the lysosome or proteasome for degradation (Scheffner et al., 1995). We predicted that the administration of UBEI-41 would reduce ubiquitylation of KCa3.1, and thus, the rate of degradation of KCa3.1 would be slowed considerably, as a result, more KCa3.1 would remain present within the cell compared to control cells. As seen in Figures 3A,B, UBEI-41 reduced the amount of degradation of KCa3.1 (*P* ≤ 0.05, *n* = 4) suggesting that an E1 ubiquitin-activating enzyme was responsible for the initial step of the ubiquitylation phase resulting in the degradation of KCa3.1. As seen at 3 h (Figure 3B), in the presence of UBEI-41, over 86 ± 6% of streptavidin-labeled KCa3.1 was retained (black bar) compared (*P* ≤ 0.05) to control cells (50 ± 8% remaining, hashed bar; control time course data from Figure 2B, *n* = 8). By 12 h, for UBEI-41-treated cells, 56 ± 7% (*P* ≤ 0.05) of KCa3.1 remained within the cell compared 5 ± 2% in control cells. Therefore, the level of the labeled channel declined slowly as incubation time increased with UBEI-41 treatment, with almost no decrease of channels after only 1 h of UBEI-41. The half-life of the channel within the cell was extended from ~3 h for control cells to beyond 12 h for UBEI-41-treated cells.

**Figure 4:**
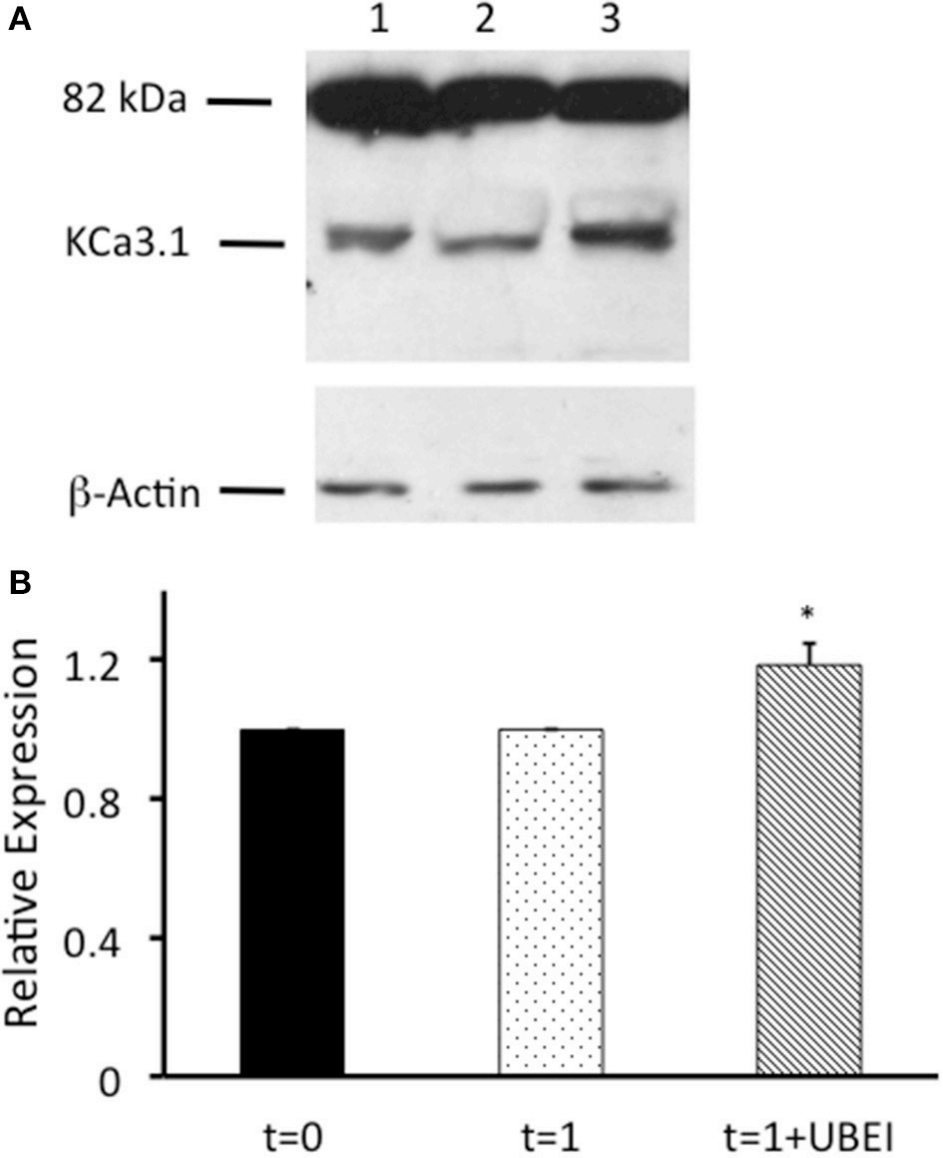
Effects of UBEI-41 on surface streptavidin labeled KCa3.1-BLAP channels. **(A)** Cells were incubated for 1 h with F12 media, or F12 media with 50 μM UBEI-41, then labeled basolaterally with streptavidin, followed by lysing and detection of KCa3.1 by immunoblot. Lane 1. KCa3.1 non-treated control cells. Lane 2: KCa3.1 1 h incubation, non-treated. Lane 3: KCa3.1 with 1 h incubation of UBEI-41. Each lane was loaded with 30 μg of protein and β-actin was used as a loading control. **(B)** Immunoblot results were quantified by densitometry. Protein expression of each time point was referenced to the control non-treated cells. Densitometry data showed that with the application of UBEI-41, the surface expression of KCa3.1 rose by 20% with 1 h treatment of UBEI-41 (hatched bar). ^*^1 h UBEI-41 treated cells exhibited a statistically significant increase in expression of KCa3.1 than the non-treated cells (dotted bar; *P* ≤ 0.05, *n* = 4).

Balut et al. (2011) and Bertuccio et al. (2014) have reported that KCa3.1 is ubiquitylated at the plasma membrane. Additionally, we have shown that the degradation of KCa3.1 is reduced in the presence of UBEI-41 (Figure 3, current study), therefore, the action of UBEI-41 may result in increased number of KCa3.1 channels being retained at the BLM. Our hypothesis was that UBEI-41 would inhibit the E1 ubiquitin-activating enzyme and disrupt ubiquitylation of the channel and endocytosis resulting in an accumulation of KCa3.1 at the BLM. Therefore, this labeling approach would provide a molecular “snapshot” of the relative amount of KCa3.1 physically present at the BLM. As shown in Figure 4, cells pretreated with UBEI-41 (50 μM, m and s; *t* = 1 h, Lane 3) exhibited a significant increase (20 ± 6%, *P* ≤ 0.05) in channels at the membrane when compared with *t* = 1 h control cells (Lane 2) or *t* = 0 h control cells (Lane 1; Figure 4B, *n* = 4). These experimental results demonstrate, for the first time in FRT cells, that UBEI-41 reduces ubiquitylation of KCa3.1 and the channel is retained at the BLM.

**Figure 5:**
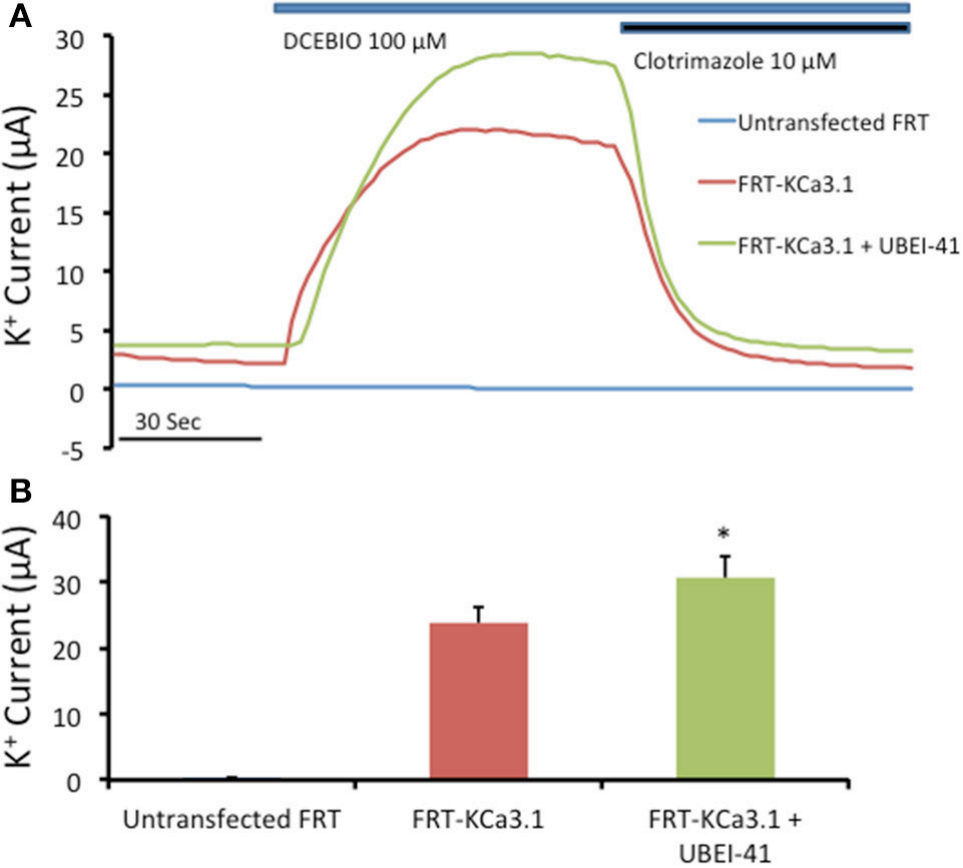
The effect of UBEI-41 on current of the KCa3.1-BLAP cells. **(A)** A representative trace of UBEI-41 treated FRT-KCa3.1-BLAP cell line. FRT-KCa3.1 cells were grown on Snapwell™ filter inserts then subjected to Ussing chamber experiments. The KCa3.1 specific activator DCEBIO (100 μM) was applied to the cells that stimulated a K^+^ current due to the opening of FRT-KCa3.1 channels. Clotrimazole (10 μM) was applied to inhibit KCa3.1 current. With the UBEI-41 (50 μM) treatment after 1 h, the DCEBIO sensitive K current was significant larger than the non-treated cells, with an averaged difference of 7 ± 1 μA. **(B)** Quantitation bar graph of the average DCEBIO sensitive K^+^ current of each tested condition. When stimulated with DCEBIO, control FRT-KCa3.1 cells exhibited an average current of 24 ± 2 μA (red bar) whereas current of UBEI-41-treated FRT-KCa3.1 cells was 31 ± 3 μA (green bar). Untransfected FRT cells did not respond to DCEBIO or clotrimazole (blue trace, Figure 5A). ^*^ UBEI-41 treated cells showed statistically significant (*P* ≤ 0.05, *n* = 4) increase of DCEBIO sensitive K^+^ current than non-treated cells.

Since, there was an increase of KCa3.1 at the BLM in the presence of UBEI-41 (50 μM, m and s; Figure 4), we predicted that if KCa3.1 is accumulated at the BLM, then, this should result in a larger I_K_ current as measured by Ussing chamber experiments. We measured the DCEBIO (100 μM, m and s)-stimulated I_K_ and the clotrimazole (10 μM, m and s)-inhibited I_K_ to determine the I_K_ attributed by KCa3.1. A representative experiment is shown in Figure 5A. As can be seen, DCEBIO stimulated a sustained I_K_ and clotrimazole inhibited the stimulated I_K_ current in both monolayers. The FRT monolayers that were incubated with UBEI-41 exhibited a significantly greater I_K_ (31 ± 3 μA, green trace and bar) compared to the untreated FRT monolayers (24 ± 2 μA, red and bar; Figure 5B; *P* ≤ 0.05, *n* = 4) providing evidence that UBEI-41 likely reduced endocytosis of ubiquitylated KCa3.1 channels from the membrane. Untransfected FRT monolayers did not respond to DCEBIO providing additional evidence of the robust stably transfected FRT cell line (Figure 5A, blue trace).

Together, these data confirm that inhibition of the E-1 ubiquitinin-activating enzyme resulted in decreased degradation of KCa3.1 in a polarized epithelium, as others have reported (Bertuccio et al., 2014). Additionally, we provide the first functional evidence that inhibition of ubiquitylation of KCa3.1 results in increased retention of KCa3.1 at the BLM and that these channels contribute to an elevated I_K_ current across the polarized epithelium. These data support the hypothesis that ubiquitin is crucial in internalization of KCa3.1, as disruption to ubiquitylation appears to impede the endocytosis of KCa3.1.

### Examination of deubiquitylation of KCa3.1 and possible deubiquitylation of membrane surface KCa3.1

**Figure 6:**
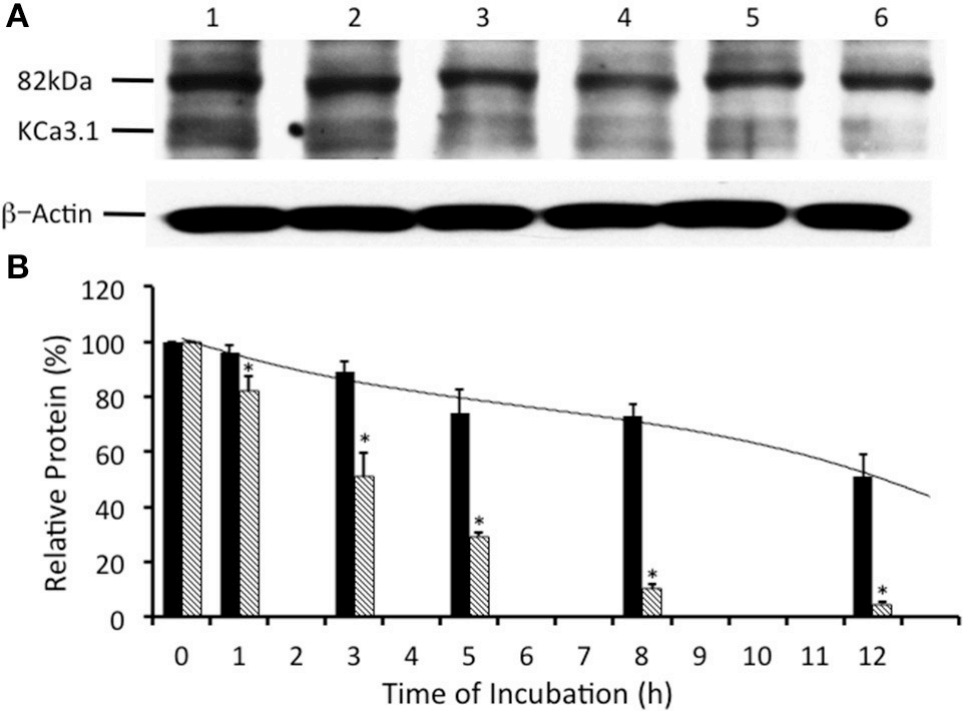
Effect of deubiquitinase inhibition via PR-619 on the streptavidin-labeled membrane bound KCa3.1-BLAP channels. **(A)** After streptavidin labeling, cells were further incubated in 37°C for 0 (no incubation), 1, 3, 5, 8, or 12 h with PR-619 (50 μM) before being lysed and detection of KCa3.1 by immunoblot. Expression level of labeled KCa3.1 declined slowly as incubation time increased, with almost no decrease of expression after 1 h of treatment. Lane 1: Control no incubation (*t* = 0), Lane 2: 1 h of incubation (*t* = 1), Lane 3: 3 h of incubation (*t* = 3), Lane 4: 5 h of incubation (*t* = 5), Lane 5: 8 h of incubation (*t* = 8), and Lane 6: 12 h of incubation (*t* = 12). Each lane was loaded with 30 μg of protein and β-actin was used as a loading control. **(B)** Immunoblot results were quantified by densitometry. Protein expression of each time point was referenced to the control non-treated, non-incubated cells. PR-619 reduced the degradation of KCa3.1 (black bars) compared to the control cells (hatched bars; data from Figure 2). Relative KCa3.1 expression, in the presence of PR-619 was similar to the expression level of KCa3.1 in the presence of UBEI-41 (Figure 3). In the presence of PR-619, nearly all of the labeled KCa3.1 remained after 1 h of treatment, and by 3 h only ~10% of the labeled channel had been degraded (black bars). Though at both the 1 and 3 h time points, there had been considerable degradation of labeled KCa3.1 of the non-treated control cells (hatched bars) (^*^P ≤ 0.05, *n* = 4). PR-619 extended the channel half-life to more than 12 h.

Previously, it has been demonstrated that KCa3.1 is ubiquitylated in both non-polarized HEK cells (Balut et al., 2011) and polarized epithelial cells Caco-2 cells (Bertuccio et al., 2014). Additionally, it has been reported that KCa3.1 expressed in HEK293 and Caco-2 cells required deubiquitylation by deubiquitylating enzymes (DUBs, proteases) that cleave ubiquitin from target proteins prior to degradation by the lysosomes or proteasomes (Martinez and Goud, 1998; Millard and Wood, 2006; Balut et al., 2011; Bertuccio et al., 2014). We wanted to determine whether KCa3.1 was deubiquitylated in our stably transfected FRT epithelial cells. We examined the effects of PR-619 (50 μM, m and s; a pan DUB inhibitor, Tian et al., 2011) on the degradation of KCa3.1. In the presence of PR-619, the degradation of KCa3.1 was reduced (Figures 6A,B). Indeed, after 5 h incubation with PR-619, channel degradation was reduced by only 25 ± 9% (*P* ≤ 0.05, *n* = 4) compared with *t* = 0. Indeed, at 5 h in control cells, KCa3.1 was significantly degraded by 70% ± 3% as compared with *t* = 0 (*P* ≤ 0.05, *n* = 8; hashed bars, Figure 6B). Here, we present a complete time course of the effect of PR-619 on the degradation rate of KCa3.1. Indeed, these data suggest that inhibition of deubiquitylation resulted in reduced degradation of KCa3.1. These data corroborate and extend, in a polarized epithelium, that KCa3.1 is deubiquitylated prior to degradation (Balut et al., 2011; Bertuccio et al., 2014).

**Figure 7:**
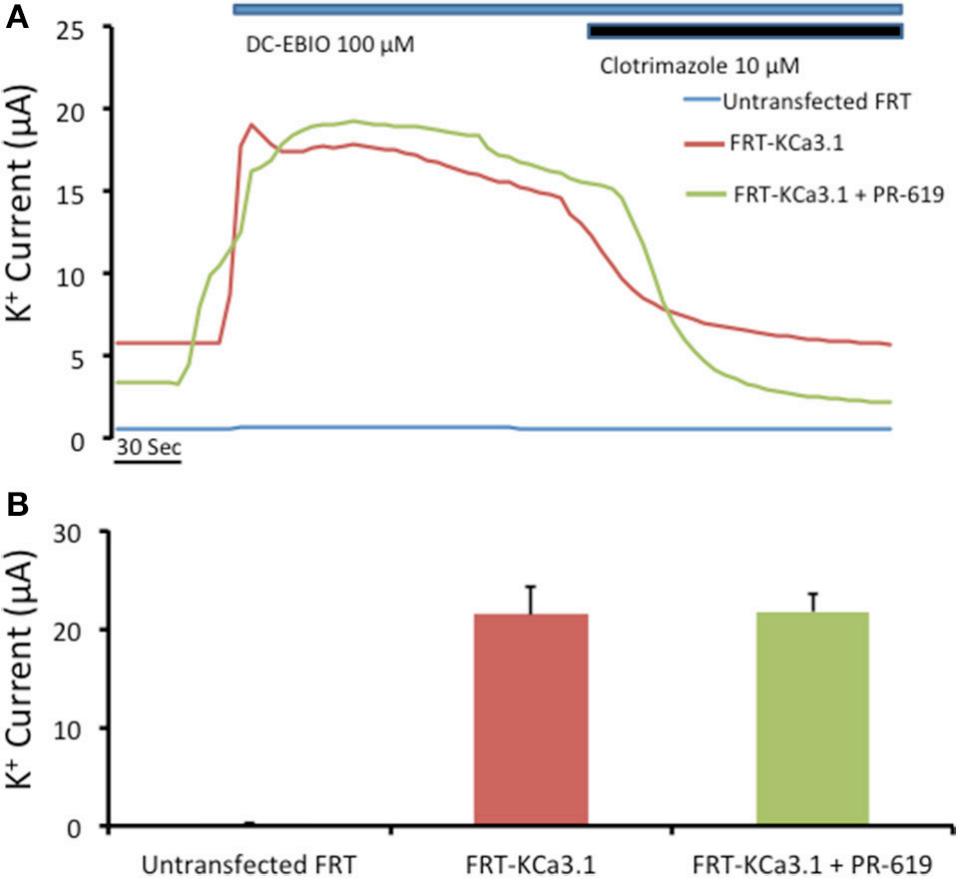
Effect of PR-619 of membrane bound FRT-KCa3.1-BLAP cells. **(A)** Representative Ussing chamber traces of the PR-619 treated cells. FRT-KCa3.1-BLAP cells were grown on Snapwell™ filter inserts then subjected to Ussing chamber experiments. Once again, DCEBIO (100 μM) were applied to the filters once the baseline current was achieved followed by clotrimazole (10 μM). After 1 h of PR-619 (50 μM) treatment (green trace), the DCEBIO sensitive K current showed no difference when compared to the untreated cells (red trace). **(B)** Quantitation bar graph of the average DCEBIO sensitive K^+^ current of each tested condition. When stimulated with DCEBIO, the untreated FRT-KCa3.1 cells exhibited an average current of 22 ± 3 μA (red bar) whereas PR-619 treated FRT-KCa3.1 cells was 22 ± 2 μA (green bar). Untransfected FRT cells did not respond to DCEBIO or clotrimazole (blue trace). Overall, cells pretreated with PR-619 exhibited the same amount of DCEBIO-stimulated I_K_ as the untreated cells (*P* > 0.05) suggesting that the effect of PR-619 was down stream of the initial ubiquitylation of membrane bound KCa3.1 (*n* = 4).

Previously, Balut et al. (2011) used a novel DUB Chip protein array approach (Loch et al., 2011) in concert with the KCa3.1-BLAP channel (Balut et al., 2010a) and determined a strong association between KCa3.1 and the DUBs USP2 and USP8 and a weak association with AMSH. Further, Balut et al. (2011) reported, using multiple experimental approaches, that USP8 regulates the delivery rate of KCa3.1 by deubiquitylating KCa3.1 prior to delivery to the lysosome. Interestingly, Verrey and colleagues (Fakitsas et al., 2007; Oberfield et al., 2011) suggested that USP2-45 deubiquitylated ENaC at the plasma membrane in concert with an SGK1-Nedd4-2 dependent mechanism leading to increased surface ENaC using a HEK293 expression system. And, as stated above, KCa3.1 appears to be directly ubiquityated at the plasma membrane (Balut et al., 2011), thus, this begs the question “Is KCa3.1 deubiquitylated at the BLM in a polarized epithelium?” Therefore, if the action of PR-619 was to prevent deubiquitylation of KCa3.1 at the BLM then we would predict that PR-619 would result in an accumulation of KCa3.1 at the BLM. It follows that, this accumulation of channels should be measured as an increased I_K_ current as measured with Ussing chamber experiments. In order to test this hypothesis, we cultured KCa3.1 cells on Snapwell™ filters for 72 h, as described above, in the absence or presence of PR-619 (50 μM, m and s) for 1 h. Untreated FRT cells grown on filters served as an overall control (Figure 7A, blue trace). A representative experiment is shown in Figure 7A. As can be noted, DCEBIO (100 μM, m and s) had a similar stimulatory effect on both KCa3.1 of FRT cells grown in the absence (22 ± 3 μA, red trace and bar) or presence of PR-619 (22 ± 2 μA, green trace and bar; Figure 7B, *n* = 4). Collectively, these data suggest that KCa3.1 is not deubiquitylated at the BLM of FRT cells. Therefore, the channel must be deubiquitylated downstream during the trafficking of the channel to the lysosome or proteasome for degradation.

### Degradation of membrane KCa3.1 by the lysosome

**Figure 8:**
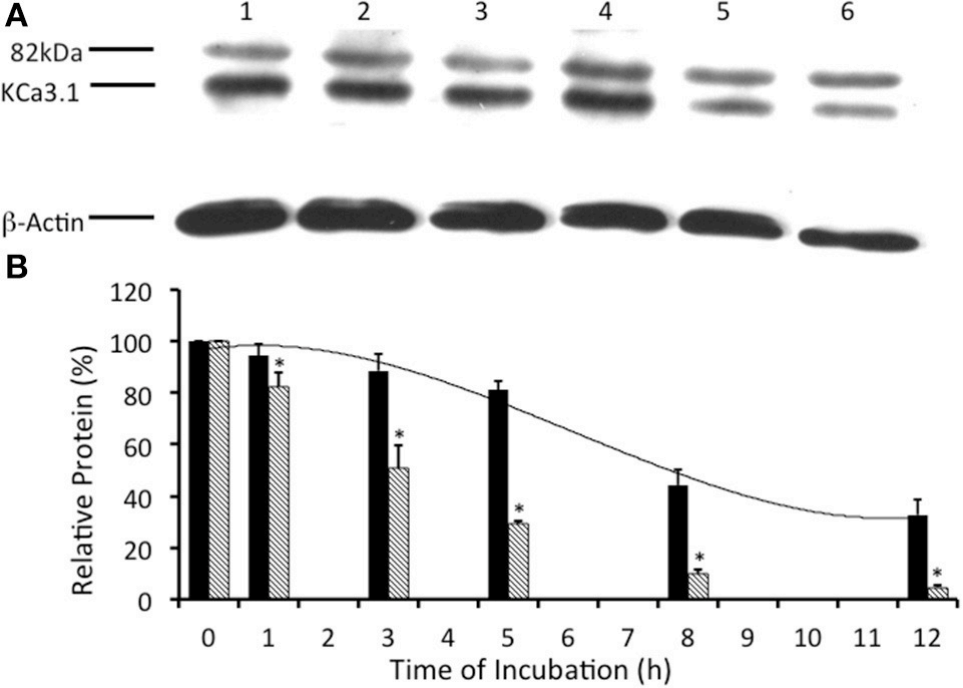
Effect of lysosomal inhibitors on the streptavidin-labeled membrane bound KCa3.1-BLAP channels. Lysosomal activities were inhibited with leupeptin (100 μM, s and m) and pepstatin (1 μg/ml, s and m; L/P) to determine the effect on the degradation of KCa3.1. **(A)** The degradation time course was executed with the addition of leupeptin and pepstatin after streptavidin labeling of KCa3.1. After labeling, cells were further incubated in 37° C for 0 (no incubation), 1, 3, 5, 8, or 12 h with L/P before cells were lysed and detection of KCa3.1 by immunoblot. Lane 1: Control no incubation (*t* = 0), Lane 2: 1 h of incubation (*t* = 1), Lane 3: 3 h of incubation (*t* = 3), Lane 4: 5 h of incubation (*t* = 5), Lane 5: 8 h of incubation (*t* = 8), and Lane 6: 12 h of incubation (*t* = 12). Each lane was loaded with 30 μg of protein and β-actin was used as a loading control. **(B)** Immunoblot results were quantified by densitometry. As seen, in the presence of L/P, the degradation of KCa3.1 (black bars) was greatly reduced, retaining high levels of KCa3.1 expressed even at 5 h after labeling compared with 5 h non-treated control cells (hatched bars) shown from Figure 2 (^*^P ≤ 0.05, *n* = 5). In the presence of L/P, the relative protein expression of KCa3.1 exhibited a half-life that was increased by ~4 h compared to normal control degradation data (Figure 2).

Degradation of cellular proteins occurs at the lysosome or the proteasome (DeMartino and Slaughter, 1999; Luzio et al., 2007). Initiation of the degradative process of membrane proteins begins with ubiquitylation of protein at the membrane followed by endocytosis. Balut et al. (2010b) determined that KCa3.1, expressed in non-polarized HEK cells, was trafficked to the lysosome for degradation by using the lysosomal protease inhibitors leupeptin and pepstatin (L/P). Additionally, Bertuccio et al. (2014) used L/P (24 h exposure) and the proteasome inhibitor lactacystin (24 h exposure) to determine that KCa3.1 was degraded at both the lysosome and proteasome in polarized Caco-2 epithelial cells. We, thus, wanted to determine a complete profile for KCa3.1 degradation by the lysosome in our stably transfected FRT cell line in the presence of leupeptin (100 μM, m and s) and pepstatin (1 μg/ml μM, m and s). As shown in Figure 8, even after 1 h, the degradation of KCa3.1 was reduced by only 5 ± 4% (*P* < 0.05) compared to control cells (18 ± 7%, *n* = 5). Indeed, when cells were incubated with L/P for 8 h, there was still only a reduction of labeled KCa3.1 by 55 ± 6% (*P* < 0.05) in the presence of L/P compared to control cells in which labeled KCa3.1 was reduced by 89 ± 2% (*n* = 5). In the presence of L/P, the time of degradation was increased to >7 h for cells treated with L/P compared to control cells of ~3 h. These results provide the first complete L/P degradation profile of KCa3.1 in polarized epithelial cells and corroborate the degradation data of KCa3.1 provided by Bertuccio et al. (2014).

## Discussion

In our current study, we investigated the modulation of retrograde trafficking of membrane surface KCa3.1 by altering ubiquitylation and deubiquitylation of KCa3.1 via pharmacological approaches. We have confirmed and extended data provided by Bertuccio et al. (2014) of KCa3.1 in polarized FRT cells and work by Balut et al. (2010b, 2011) conducted in non-polarized HEK cells. Here, we have demonstrated that KCa3.1 is targeted exclusively to the BLM, have provided a complete time course of degradation of KCa3.1 and time courses of the channel in the presence of pharmacological inhibitors of ubiquitylation and deubiquitylation to advance our understanding of the retrograde trafficking of KCa3.1. Further, we demonstrated that inhibition of ubiquitylation of membrane bound KCa3.1 resulted in increased numbers of channels remaining at the BLM and which contribute to physiological responses (Figures 4, 5). Additionally, we have provided the first functional evidence that KCa3.1 is not deubiquitylated at the BLM and that deubiquitylation must occur further downstream in the degradation process of KCa3.1 (Figures 6, 7).

Interestingly, the time course of degradation of plasma membrane KCa3.1 varies from 3 to ~16 h depending upon the heterologous cell expression system (HEK, Caco-2, MDCK, and FRT) and cell cultures conditions (impermeable, plastic, vs. permeable supports) resulting in non-polarized or polarized epithelia (Balut et al., 2010a, 2011; Bertuccio et al., 2014). In our study, membrane labeled KCa3.1 exhibited a half-life for degradation of ~3 h in polarized FRT cells (Figure 2), similar to data reported by Jones et al. (2004) for cellular KCa3.1 expressed in HEK cells cultured on plastic. Bertuccio et al. (2014) reported that surface labeled KCa3.1 expressed in Caco-2, MDCK, and FRT cells (on permeable filters) exhibited a half-life for degradation of >16, >5, and ~3 h, respectively. Additionally, endocytosed labeled channels were still identified by IB at 30 h for Caco-2 cells.

There is ongoing debate whether KCa3.1 is trafficked directly to the cell membrane or trafficked via a recycling endosomal pathway. KCa2.3 (KCNN3), another member of the KCNN gene family, exhibited a long membrane residence time when expressed in HEK cells and human microvascular endothelial cells with a half-life of degradation of >18 h (Gao et al., 2010). Gao et al. (2010) utilized a membrane channel stripping protocol to conclusively demonstrate that KCa2.3, expressed in HEK cells, was quickly endocytosed and recycled back to the plasma membrane. In contrast, Bertuccio et al. (2014) exploited the fact that the transferrin receptor (TfnR) is recycled, via the recycling endosome, back to the plasma membrane (Maxfield and McGraw, 2004) to determine whether KCa3.1 utilized a recycling endosomal pathway to the BLM. Bertuccio et al. (2014) provided the first evidence that KCa3.1 does not use a recycling endosome when trafficking to the membrane by transducing both the TfnR and KCa3.1-BLAP in MDCK cells and demonstrating that KCa3.1 trafficked to the plasma membrane in both control and recycling endosome ablated cells. Thus, KCa3.1 was trafficked directly to the BLM and not via a recycling endosome pathway used by TfnR. From our study, the only suggestive evidence that the channel does not recycle is that the channel has a short half-life so it appears unlikely that channels spend a lot of time recycling. Maylie et al. (Lin et al., 2012), using patch-clamp experiments, reported in endothelial cells that unlike KCa2.3, KCa3.1 did not utilize caveolae-mediated membrane cycling and that KCa3.1 did not undergo dynamic cycling. Additionally, Gao et al. (2010) reported that KCa3.1 was quickly endocytosed and degraded and did not enter a recycling compartment providing added evidence that KCa3.1 may not recycle. Finally, Schwab et al. (2012) demonstrated, in migrating MDCK-F cells, that KCa3.1 exhibited clathrin-dependent endocytosis during cell migration and that the authors observed that KCa3.1 containing vesicles were newly formed at the plasma membrane and those vesicles were optically tracked to the leading edge of the lamellipodium of the migrating cells, suggesting the recycling of KCa3.1 to the plasma membrane. It would have been useful if the authors had executed a recycling assay of membrane bound channels in order to directly demonstrate recycling of KCa3.1. Clearly, further studies are required to define whether KCa3.1 recycles or not.

### Modulation of ubiquitylation of KCa3.1

Ubiquitylation of a membrane surface protein serves as a signal for endocytosis via clathrin-coated vesicles after which the protein can be recycled back to the membrane or traverse via the ESCRT pathway to the lysosome (Clague et al., 2012). Balut et al. (2010a) provided the first evidence that surface KCa3.1 was ubiquitylated within 90 min. We have extended that finding and have provided the first complete degradative profile of KCa3.1 in the presence of UBEI-41 (an E1-activating enzyme inhibitor) in polarized epithelial cells where the rate of channel degradation was significantly reduced (56 ± 7% remaining at 12 h) compared with control cells (5 ± 2% remaining at 12 h; Figure 3). Balut et al. (2011) demonstrated that KCa3.1 was only slightly ubiquitylated at the membrane, however, significant ubiquitylation of KCa3.1 occurred after the channels were endocytosed in unpolarized HEK cells. Similarly, Bertuccio et al. (2014) reported that a small amount of KCa3.1 was ubiquitylated at the membrane, however a further ubiquitylation of KCa3.1 followed endocytosis in polarized Caco-2 cells.

Interestingly, we observed that when we incubated FRT cells in the presence of UBEI-41 for 1 h, 96 ± 2% of KCa3.1 was retained in the cells compared with 82 ± 5% retained KCa3.1 was observed in untreated control cells (Figure 3). Based on those results, we, next, asked the question “Would inhibiting the E1-activating enzyme by UBEI-41 result in an increased amount of KCa3.1 retained at the BLM?” Therefore, we predicted that if UBEI-41 prevented ubiquitylation of surface bound KCa3.1 there might be an accumulation of KCa3.1 at the membrane that could be identified by IB and Ussing experiments. Here, we provide the first IB (Figure 4) and functional (Ussing experiments, Figure 5) evidence that demonstrated that UBEI-41 resulted in the retention of KCa3.1 at the BLM. As measured by IB, the effect of 1 h exposure of cells with UBEI-41 increased KCa3.1 at the BLM compared to control cells (Figure 4). Further, cells incubated with UBEI-41 for 1 h exhibited an increased DCEBIO-sensitive, clotrimazole-sensitive I_K_ current hallmark of KCa3.1 compared to currents of control cells (Figure 5).

### Is KCa3.1 deubiquitylated at the basolateral membrane?

The mode of action of DUBs is varied including the classical deubiquitylation of ubiquityated proteins within late endosomes destined for degradation by the lysosome or proteasome (Clague et al., 2012, 2013). Indeed, it is thought that proteins targeted for degradation may be deubiquitylated prior to arriving at the lysosome to recycle cellular ubiquitin (Reyes-Turcu et al., 2009). In fact, Balut et al. (2011) examined the deubiquitylation of KCa3.1 prior to lysosomal delivery and demonstrated that the DUB inhibitor PR-619 slowed degradation of KCa3.1 (in non-polarized HEK cells) resulting in over 85% of the labeled membrane channel was still observed within the cell at 8 h while only ~45% of the channel remained present in control cells. Here, we provided the first complete experimental time course of the effects of PR-619 on the deubiquitylation of KCa3.1 in polarized epithelial FRT cells (Figure 6). Similar to Balut et al. (2011), at 8 h incubation of PR619, that 75 ± 8% of membrane-labeled KCa3.1 still remained within the FRT cells compared to 15 ± 2% for KCa3.1 of control cells. Also, after 12 h treatment of PR619, over 50% of the membrane-labeled KCa3.1 still remained within the cells compared to 8 ± 2% for control cells (Figure 6). Similarly, Bertuccio et al. (2014) reported that PR-619 reduced the degradation of KCa3.1 transduced into Caco-2 cells where 87 ± 1% of KCa3.1 was retained after 24 h while in control Caco-2 cells only 24 ± 1% of KCa3.1 was retained. It must be noted that KCa3.1 transduced in to Caco-2 cells has a much longer half-life (>12 h) compared with KCa3.1 expressed in FRT cells (Figure 2, this study) and MDCK cells (Bertuccio et al., 2014). At present, we still do not understand the intricate dynamics of this process in various cell systems.

Even though deubiquitylation of proteins is thought to occur in the vicinity of the lysosome, there is growing evidence suggesting that DUBs play important roles in modulating plasma membrane proteins numbers by deubiquitylating membrane proteins (i.e., ion channels/transporters and receptors) within early endosomes that are then recycled back to the plasma membrane (Clague et al., 2013). Indeed, Butterworth et al. (2007) and Bomberger et al. (2009, 2010) demonstrated for ENaC and CFTR, respectively, that the DUBs UCH-L3 for ENaC and USP10 for CFTR play vital roles in the recycling of these channels back to the apical membrane of epithelial cells. Further, it has been demonstrated that some DUBs can deubiquityate membrane resident proteins. For example, Verrey and colleagues (Fakitsas et al., 2007; Oberfield et al., 2011) used a HEK293 expression system to demonstrate that UPS2-45 deubiquitylates ENaC at the plasma membrane in concert with a SGK1-Nedd4-2 dependent mechanism. Also, Seki et al. (2013) reported that the membrane-localized DUB, JosD1, transfected in HEK cells, deubiquitylated membrane receptors after agonist stimulation leading to a decrease in clathrin- and caveolae-mediated endocytosis, thus maintaining receptors at the plasma membrane. It has been suggested that KCa3.1 is endocytosed by clathrin-mediated endocytosis in migrating cells (Schwab et al., 2012). As shown in Figure 6, PR-619 increased the amount of channel remaining in the cell at *t* = 1 h and also increased the half-life of the labeled channels (>12 h) as compared to control cells (~3 h). Therefore, the increased amount of KCa3.1 in the presence of PR-619 retained after 1 h may be due to a reduction in clathrin-dependent endocytosis and/or deubiquitylation of membrane bound ubiquitylated KCa3.1. If the later was the reason for the retained KCa3.1 then we would hypothesized that cells incubated with PR-619 would generate a higher I_K_ current than untreated cells as measured with Ussing experiments. This hypothesis was disproven as the I_K_ current exhibited by PR-619-treated cells (*t* = 1 h) was no different from the I_K_ of untreated cells (Figure 7). Therefore, the effect of PR-619 on KCa3.1 must occur downstream of the endocytosis step of the channel. Further experiments are required to tease apart the location of the deubiquitylation of KCa3.1 prior to degradation by the lysosome.

### KCa3.1 as a therapeutic target?

KCa3.1 has been purported to be a therapeutic target in a number of diseases (Köhler, 2009; Köhler et al., 2010; Wulff and Castle, 2010). Indeed, changes in the membrane resident population of KCa3.1 can lead to differences in overall physiological function. For example, Ghanshani et al. (2000) reported that the numbers of KCa3.1 channels were increased by mitogens resulting in the activation of human naïve T-lymphocytes via the transcription factors, AP1 and Ikaros-2. Using colon from KCa3.1 null mice, Flores et al. (2007) demonstrated that heterozygous mice (+/−) exhibited only 40% of the histamine- and carbachol-stimulated of the Cl^−^ secretory response compared with that of WT (+/+) mice as measured with Ussing chamber experiments. Lastly, Al-Hazza et al. (2012) reported that there were decreased numbers of KCa3.1 channels in active UC patients, though similar number of channels of UC patients whom were in remission compared to control subjects. These data suggest that the number or activity of the KCa3.1 channels at the membrane is critical for physiological function. Nonetheless, our data provide a glimpse of the pharmacological modulation of this channel in an epithelial expression system. It would be most challenging to extrapolate potential therapeutic avenues of KCa3.1 at this point with a pharmacological approach.

In summary, we have provided complete time course experiments examining the effects of pharmacological modulators of ubiquitylation and deubiquitylation of KCa3.1 in polarized epithelial cells. Additionally, we provide the first functional evidence that ubiquitylated channels are retained at the BLM and that deubiquitylation of KCa3.1 appears not to occur at the BLM of FRT cells.

## Author contributions

KH designed the experiments with consultation of BL and DD. BL conducted all the experiments. KH and BL interpreted the experimental results in consultation with DD. BL conducted the statistical analyses with advice from KH. All authors approved the final version of the manuscript.

### Conflict of interest statement

The authors declare that the research was conducted in the absence of any commercial or financial relationships that could be construed as a potential conflict of interest.

## References

[B1] Albaqumi M. Strivastava S. Li Z. Zhdnova O. Wulff H. Itani O.. (2008). KCa3.1 potassium channels are critical for cAMP-dependent chloride secretion and cyst growth in autosomal-dominant polycystic kidney disease. Kidney Int. 74, 740–749. 10.1038/ki.2008.246 18547995

[B2] Al-Hazza A. Linley J. E. Aziz Q. Maclennan K. A. Hunter M. Sandle G. I. (2012). Potential role of reduced basolateral potassium (KCa3.1) channel expression in the pathogenesis of diarrhea in ulcerative colitis. J. Pathol. 226, 463–470. 10.1002/path.2994 22009605

[B3] Andolfo A. Russo R. Manna F. Shmukler B. E. Gambale A. Vitiello G.. (2015). Novel Gardos channel mutations linked to dehydrated hereditary stomatocytosis (xerocytosis). Am. J. Hematol. 90, 921–927. 10.1002/ajh.24117 26178367

[B4] Balut C. M. Gao Y. Luke C. Devor D. C. (2010a). Immunofluorescence-based assay to identify modulators of the number of plasma membrane KCa3.1 channels. Future Med. Chem. 2, 707–713. 10.4155/fmc.10.182 20596245 PMC2892982

[B5] Balut C. M. Gao Y. Murray S. A. Thibodeau P. H. Devor D. C. (2010b). ESCRT-dependent targeting of plasma membrane localized KCa3.1 to the lysosomes. Am. J. Physiol. Cell Physiol. 299, C1015–C1027. 10.1152/ajpcell.00120.2010 20720181 PMC2980317

[B6] Balut C. M. Hamilton K. L. Devor D. C. (2012). Trafficking of intermediate (KCa3.1) and small (KCa2.x) conductance, Ca^2+^-activated K^+^ channels: a novel target for medicinal chemistry efforts? ChemMedChem 7, 1741–1755. 10.1002/cmdc.201200226 22887933 PMC3455125

[B7] Balut C. M. Loch C. M. Devor D. C. (2011). Role of ubiquitylation and USP8-dependent deubiquitylation in the endocytosis and lysosomal targeting of plasma membrane KCa3.1. FASEB J. 25, 3938–3948. 10.1096/fj.11-187005 21828287 PMC3205838

[B8] Bertuccio C. A. Lee S.-L. Wu G. Butterworth M. B. Hamilton K. L. Devor D. C. (2014). Anterograde trafficking of KCa3.1 in polarized epithelia is Rab1- and Rab8-dependent and recycling endosomal-independent. PLoS ONE 9:e92013 10.1371/journal.pone.0092013 24632741 PMC3954861

[B9] Bomberger J. M. Barnaby R. L. Stanton B. A. (2009). The deubiquitinating enzyme USP10 regulates the post-endocytolic sorting of cystic fibrosis transmembrane conductance regulator in airway epithelial cells. J. Biol. Chem. 284, 18778–18789. 10.1074/jbc.M109.001685 19398555 PMC2707225

[B10] Bomberger J. M. Barnaby R. L. Stanton B. A. (2010). The deubiquitinating enzyme USP10 regulates the endocytic recycling of CFTR in airway epithelial cells. Channels 4, 150–154. 10.4161/chan.4.3.11223 20215869 PMC3678275

[B11] Butterworth M. B. Edinger R. S. Ovaa H. Burg D. Johnson J. P. Frizzell R. A. (2007). The deubiquitinating enzyme UCH-L3 regulates the apical membrane recycling of the epithelial sodium channel. J. Biol. Chem. 282, 37885–37893. 10.1074/jbc.M707989200 17967898

[B12] Chachi L. Shikotra A. Duffy S. M. Tliba O. Brightling C. Bradding P. (2013). Function K_*Ca*_3.1 channels regulate steroid insensitivity in bronchial smooth muscle cells. J. Immunol. 191, 2624–2636. 10.4049/jimmunol.1300104 23904164 PMC3753579

[B13] Chen I. Howarth M. Lin W. Ting A. L. (2005). Site-specific labeling of cell surface proteins with biophysical probes using biotin ligase. Nat. Methods 2, 99–104. 10.1038/nmeth735 15782206

[B14] Clague M. J. Liu H. Urbé S. (2012). Governance of endocytic trafficking and signaling by reversible ubiquitylation. Develop. Cell 23, 457–467. 10.1016/j.devcel.2012.08.011 22975321

[B15] Clague M. L. Barsukov I. Coulson J. M. Liu H. Rigden D. J. Urbé S. (2013). Deubiquitylases from genes to organism. Physiol. Rev. 93, 1289–1315. 10.1152/physrev.00002.2013 23899565

[B16] DeMartino G. N. Slaughter C. A. (1999). The proteasome, a novel protease regulate by multiple mechanisms. J. Biol. Chem. 274, 22123–22126. 10.1074/jbc.274.32.22123 10428771

[B17] Devor D. C. Bertuccio C. A. Hamilton K. L. (2016). KCa3.1 in epithelia, Chapter 20, in Ion Channels and Transporters of Epithelia in Health and Disease, eds Hamilton K. L. Devor D. C. (New York, NY: Springer-Verlag), 659–705.

[B18] Devor D. C. Frizzell R. A. (1993). Calcium-mediated agonists activate an inwardly rectified K^+^ channel in colonic secretory cells. Am. J. Physiol. Cell Physiol. 265, C1271–C1280. 7694492 10.1152/ajpcell.1993.265.5.C1271

[B19] Devor D. C. Singh A. K. Gerlach A. C. Frizzell R. A. Bridges R. J. (1997). Inhibition of intestinal Cl^−^ secretion by clotrimazole: direct effect on basolateral membrane K^+^ channels. Am. J. Physiol. Cell Physiol. 273, C531–C540. 9277350 10.1152/ajpcell.1997.273.2.C531

[B20] Durham A. C. (1983). A survey of readily available chelators for buffering calcium ion concentrations in physiological solutions. Cell Calcium 4, 33–46. 10.1016/0143-4160(83)90047-7 6682712

[B21] Fakitsas P. Adam G. Daidié D. van Bemmelen M. X. Fouladkou F. Patrignani A.. (2007). Early aldosterone-induced gene product regulates the epithelial sodium channel by deubiquitylation. J. Am. Soc. Nephrol. 18, 1084–1092. 10.1681/ASN.2006080902 17344426

[B22] Farquhar R. Rodriques E. Hamilton K. L. (2017). The role of the cytoskeleton and Myosin-Vc in the targeting of Kca3.1 to the basolateral membrane of polarized epithelial cells. Front. Physiol. 7:639. 10.3389/fphys.2016.00639 28101059 PMC5209343

[B23] Flores C. A. Melvin J. E. Figueroa C. D. Sepúlveda F. V. (2007). Abolition of Ca^2+^-mediated intestinal anion secretion and increased stool dehydration in mice lacking the intermediate conductance Ca^2+^-dependent K^+^ channel Kcnn4. J. Physiol. (Lond). 583, 705–717. 10.1113/jphysiol.2007.134387 17584847 PMC2277011

[B24] Gao Y. Balut C. M. Bailey M. A. Patino-Lopez G. Shaw S. Devor D. C. (2010). Recycling of the Ca^2+^-activated K^+^ channel, KCa2.3, is dependent upon RME-1, Rab35/EPI64C, and an N-terminal domain. J. Biol. Chem. 285, 17938–17953. 10.1074/jbc.M109.086553 20360009 PMC2878556

[B25] Gao Y. Chotoo C. K. Balut C. M. Sun F. Bailey M. A. Devor D. C. (2008). Role of S3 and S4 transmembrane domain charged amino acids in channel biogenesis and gating of KCa2.3 and KCa3.1. J. Biol. Chem. 283, 9049–9059. 10.1074/jbc.M708022200 18227067 PMC2431042

[B26] Ghanshani S. Wulff H. Miller M. J. Rohm H. Neben A. Gutman G. A.. (2000). Up-regulation of the *IKCa1* potassium channel during T-cell activation. J. Biol. Chem. 275, 37137–37149. 10.1074/jbc.M003941200 10961988

[B27] Glogowska E. Lezon-Geyda K. Maksimova Y. Schultz V. P. Gallagher P. G. (2015). Mutations in the Gardos channel (*KCNN4*) are associated with hereditary xerocytosis. Blood 126, 1281–1284. 10.1182/blood-2015-07-657957 26198474 PMC4566808

[B28] Hamilton K. L. Keissling M. (2006). DCEBIO stimulates Cl^−^ secretion in the mouse jejunum. Am. J. Physiol. Cell Physiol. 290, C152–C164. 10.1152/ajpcell.00187.2005 16135545

[B29] Hamilton K. L. Meads L. Butt A. G. (1999). 1-EBIO stimulates Cl^−^ secretion by activating a basolateral K^+^ channel in the mouse jejunum. Pfluegers Arch. 439, 158–166. 10.1007/s004249900137 10651013

[B30] Howarth M. Ting A. L. (2008). Imaging proteins in live mammalian cells with biotin ligase and monovalent streptavidin. Nat. Protocols 8, 534–545. 10.1038/nprot.2008.20 PMC2671200 18323822

[B31] Ishii T. M. Silvia C. Hirschberg B. Bond C. T. Adelman J. P. Maylie J. (1997). A human intermediate conductance calcium-activated potassium channel. Proc. Natl. Acad. Sci. U.S.A. 94, 11651–11656. 10.1073/pnas.94.21.11651 9326665 PMC23567

[B32] Joiner W. J. Wang L.-Y. Tang M. D. Kaczmarek L. K. (1997). hSK4, a member of a novel subfamily of calcium-activated potassium channels. Proc. Natl. Acad. Sci. U.S.A. 94, 11013–11018. 10.1073/pnas.94.20.11013 9380751 PMC23566

[B33] Jones H. M. Bailey M. A. Baty C. J. Macgregor G. G. Syme C. A. Hamilton K. L.. (2007). An NH_2_-terminal multi-basic RKR motif is required for the ATP-dependent regulation of hIK1. Channels (Austin) 1, 80–91. 10.4161/chan.3999 18690018 PMC3419011

[B34] Jones H. M. Hamilton K. L. Devor D. C. (2005). Role of an S4-S5 linker lysine in the trafficking of the Ca^2+^-activated K^+^ channels IK1 and SK3. J. Biol. Chem. 280, 37257–37265. 10.1074/jbc.M508601200 16135513

[B35] Jones H. M. Hamilton K. L. Papworth G. D. Syme C. A. Watkins S. C. Bradbury N. A.. (2004). Role of the NH_2_ terminus in the assembly and trafficking of the intermediate conductance Ca^2+^-activated K^+^ channel hIK1. J. Biol. Chem. 279, 15531–15540. 10.1074/jbc.M400069200 14754884

[B36] Köhler R. (2009). Single-nucleotide polymorphisms in vascular Ca^2+^-activated K^+^-channel genes and cardiovascular disease. Pflügers Arch. 460, 343–351. 10.1007/s00424-009-0768-6 20043229

[B37] Köhler R. Degenhardt C. Kuhn M. Runkel N. Paul M. Hoyer J. (2000). Expression and function of endothelial Ca^2+^-activated K^+^ channels in human mesenteric artery. A single-cell reverse transcriptase–polymerase chain reaction and electrophysiological study *in situ*. Circ. Res. 87, 496–503. 10.1161/01.RES.87.6.496 10988242

[B38] Köhler R. Kaistha B. P. Wulff H. (2010). Vascular KCa-channels as therapeutic targets in hypertension and restenosis disease. Expert Opin. Ther. Targets 14, 143–155. 10.1517/14728220903540257 20055714 PMC3644209

[B39] Lin M. T. Adelman J. P. Maylie J. (2012). Modulation of endothelial SK3 channel activity by Ca^2+^-dependent cavelolar trafficking. Am. J. Physiol. Cell Physiol. 303, C318–C327. 10.1152/ajpcell.00058.2012 22621787 PMC3423019

[B40] Loch C. M. Cuccherini C. L. Leach C. A. Stickler J. E. (2011). Deubiquitylase, DeSUMOylase, and DelSGylase activity microarrays for assay of substrate preference and functional modifiers. Mol. Cell. Proteomics 10:M110.002402. 10.1074/mcp.M110.002402 20956615 PMC3013450

[B41] Logsdon N. J. Kang J. Togo J. A. Christian E. P. Aiyar J. (1997). A novel gene, hKCa4, encodes the calcium-activated potassium channel in human T lymphocytes. J. Biol. Chem. 272, 32723–32726. 10.1074/jbc.272.52.32723 9407042

[B42] Luzio J. P. Pryor P. R. Bright N. A. (2007). Lysosomes: fusion and function. Nat. Rev. Mol. Cell Biol. 8, 622–632. 10.1038/nrm2217 17637737

[B43] Martinez O. Goud B. (1998). Rab proteins. Biochim. Biophys. Acta 1404, 101–112. 10.1016/S0167-4889(98)00050-0 9714762

[B44] Maxfield F. R. McGraw T. E. (2004). Endocytic recycling. Nat. Rev. Mol. Cell Biol. 5, 121–132. 10.1038/nrm1315 15040445

[B45] McCann J. D. Welsh M. J. (1990). Basolateral K^+^ channels in airway epithelia II. Role in Cl^−^ secretion and evidence for two types of K^+^ channel. Am. J. Physiol. Lung Cell Mol. Physiol. 258, L343–L348. 1694405 10.1152/ajplung.1990.258.6.L343

[B46] Millard S. M. Wood S. A. (2006). Riding the DUBway: regulation of protein trafficking by deubiquitylating enzymes. J. Cell Biol. 173, 463–468. 10.1083/jcb.200602082 16702236 PMC2063856

[B47] Neutzner M. Neutzner A. (2012). Enzymes of ubiquitination and deubiquitination. Essays Bioc. 52, 37–50. 10.1042/bse0520037 22708562

[B48] Neylon C. B. Avdonin P. V. Larsen M. A. Bobik A. (1994). Rat aortic smooth muscle cells expressing charybdotoxin-sensitive potassium channels exhibit enhanced proliferative responses. Clin. Exp. Pharmacol. Physiol. 21, 117–120. 10.1111/j.1440-1681.1994.tb02477.x 7518756

[B49] Neylon C. B. Lang R. J. Fu Y. Bobik A. Reinhart P. H. (1999). Molecular cloning and characterization of the intermediate-conductance Ca^2+^-activated K^+^ channel in vascular smooth muscle: relation between K_*Ca*_ channel diversity and smooth muscle cell function. Circ. Res. 29, e33–43. 10.1161/01.res.85.9.e33 10532960

[B50] Neylon C. B. Nurgali K. Hunne B. Robbins H. L. Moore S. Chen M. X.. (2004). Intermediate-conductance calcium-activated potassium channels in enteric neurones of the mouse: pharmacological, molecular and immunochemical evidence for their role in mediating the slow afterhyperpolarization. J. Neurochem. 90, 1414–1422. 10.1111/j.1471-4159.2004.02593.x 15341525

[B51] Oberfield B. Ruffieux-Daidié D. Vitagliano J. J. Pos K. M. Verrey F. Staub O. (2011). Ubiquitinin-specific protease 2-45 (Usp2-45) binds to epithelial Na^+^ channel (ENaC)-ubiquitylating enzyme Nedd4-2. Am. J Physiol. Renal. Physiol. 301, F189–F196. 10.1152/ajprenal.00487.2010 21478478

[B52] Rapetti-Mauss R. Lacoste C. Picard V. Guitton C. Lombard E. Loosveld M.. (2015). A mutation in the Gardos channel is associated with hereditary xerocytosis. Blood 126, 1273–1280. 10.1182/blood-2015-04-642496 26148990

[B53] Reyes-Turcu F. E. Ventii K. H. Wilkinson K. D. (2009). Regulation and cellular roles of ubiquitin-specific deubiquitinating enzymes. Annu. Rev. Biochem. 78, 363–397. 10.1146/annurev.biochem.78.082307.091526 19489724 PMC2734102

[B54] Rotin D. Staub O. (2011). Role of the ubiquitin system in regulating ion transport. Pflügers Arch. 461, 1–21. 10.1007/s00424-010-0893-2 20972579

[B55] Scheffner M. Nuber U. Huibregste J. M. (1995). Protein ubiquitination involving an E1-E2-E3 emzyme ibiquitin thioester cascade. Nature 373, 81–85. 10.1038/373081a0 7800044

[B56] Schwab A. Nechyporuk-Zloy V. Gassner B. Schulz C. Kessler W. Mally S. (2012). Dynamic redistribution of calcium sensitive potassium channels (hK_*Ca*_3.1) in migrating cells. J. Cell. Physiol. 227, 686–696. 10.1002/jcp.22776 21465474

[B57] Seki T. Gong L. Williams A. J. Sakai N. Todi S. Paulson H. L. (2013). JosD1, a membrane-targeted deubiquitinating enzyme, is activated by ubiquitination and regulates membrane dynamics, cell motility and endocytosis. J. Biol. Chem. 288, 17145–17155. 10.1074/jbc.M113.463406 23625928 PMC3682520

[B58] Singh S. Syme C. A. Singh A. K. Devor D. C. Bridges R. J. (2001). Benzimidazolone activators of chloride secretion: potential therapeutics for cystic fibrosis and chronic obstructive pulmonary disease. J. Pharmacol. Exp. Ther. 296, 600–611. 11160649

[B59] Syme C. A. Hamilton K. L. Jones H. M. Gerlach A. C. Giltinan L. Papworth G. D.. (2003). Trafficking of the Ca^2+^-activated K^+^ channel, hIK1, is dependent upon a C-terminal leucine zipper. J. Biol. Chem. 278, 8476–8486. 10.1074/jbc.M210072200 12493744

[B60] Tian X. Isamiddinova N. S. Peroutka R. J. Goldenberg S. J. Mattern M. R. Nicholson B.. (2011). Characterization of selective ubiquitin and ubiquitin-like protease inhibitors using a fluorescence-based multiplex assay format. Assay Drug Dev. Technol. 9, 165–173. 10.1089/adt.2010.0317 21133675 PMC3065724

[B61] Turner R. W. Kruskic M. Teves M. Scheidl-Yee T. Hameed S. Zamponi G. W. (2015). Neuronal expression of the intermediate conductance calcium-activated potassium channel KCa3.1 in the mammalian central nervous system. Pflügers Arch. 467, 311–328. 10.1007/s00424-014-1523-1 24797146

[B62] Walker J. M. (1994). The bicinchoninic acid (BCA) assay for protein quantitation. Meth. Mol. Biol. 32, 5–8. 10.1385/0-89603-268-x:5 7951748

[B63] Weisbrod D. Peretz A. Ziskind A. Menaker N. Oz S. Barad L.. (2013). SK4 Ca^2+^ activated K^+^ channel is a critical player incardiac pacemaker derived from human embryonic stem cells. Proc. Natl. Acad. Sci. U.S.A. 110, E1685–E1694. 10.1073/pnas.1221022110 23589888 PMC3645592

[B64] Winter B. Balut C. M. Butterworth M. B. Gao Y. Devor D. C. Hamilton K. L. (2011). Basolateral trafficking of KCa3.1 in a polarized epithelium. FASEB J. 25, 860.813.

[B65] Wulff H. Castle N. A. (2010). Therapeutic potential of KCa3.1 blockers: recent advances and promising trends. Expert Rev. Clin. Pharmacol. 3, 385–396. 10.1586/ecp.10.11 22111618 PMC3347644

[B66] Yang Y. Kitagaki J. Dai R. M. Tsai Y. C. Lorick K. L. Ludwig R. L.. (2007). Inhibitors of ubiquitin-activating enzyme (E1), a new class of potential cancer therapeutics. Cancer Res. 67, 9472–9481. 10.1158/0008-5472.CAN-07-0568 17909057

[B67] Zurzolo C. Gentile R. Mascia A. Garbi C. Polistina C. Aloj L.. (1991). The polarized epithelial phenotype is dominant in hybrids between polarized and unpolarized rat thyroid cell lines. J. Cell Sci. 98, 65–73. 1711531 10.1242/jcs.98.1.65

